# Differential expression of *Spiroplasma citri* surface protein genes in the plant and insect hosts

**DOI:** 10.1186/s12866-016-0666-y

**Published:** 2016-03-22

**Authors:** Marie-Pierre Dubrana, Laure Béven, Nathalie Arricau-Bouvery, Sybille Duret, Stéphane Claverol, Joël Renaudin, Colette Saillard

**Affiliations:** UMR 1332 Biologie du Fruit et Pathologie, INRA, F-33882 Villenave d’Ornon, France; UMR 1332 Biologie du Fruit et Pathologie, Université de Bordeaux, F-33882 Villenave d’Ornon, France; Plateforme Protéome, CGFB, Université de Bordeaux, F-33076 Bordeaux, France

**Keywords:** Spiroplasma, *Spiroplasma citri*, Lipoproteins, Adhesins, Gene expression

## Abstract

**Background:**

*Spiroplasma citri* is a cell wall-less, plant pathogenic bacteria that colonizes two distinct hosts, the leafhopper vector and the host plant. Given the absence of a cell wall, surface proteins including lipoproteins and transmembrane polypeptides are expected to play key roles in spiroplasma/host interactions. Important functions in spiroplasma/insect interactions have been shown for a few surface proteins such as the major lipoprotein spiralin, the transmembrane *S. citri* adhesion-related proteins (ScARPs) and the sugar transporter subunit Sc76. *S. citri* efficient transmission from the insect to the plant is expected to rely on its ability to adapt to the different environments and more specifically to regulate the expression of genes encoding surface-exposed proteins.

**Results:**

Genes encoding *S. citri* lipoproteins and ScARPs were investigated for their expression level in axenic medium, in the leafhopper vector *Circulifer haematoceps* and in the host plant (periwinkle *Catharanthus roseus*) either insect-infected or graft-inoculated. The vast majority of the lipoprotein genes tested (25/28) differentially responded to the various host environments. Considering their relative expression levels in the different environments, the possible involvement of the targeted genes in spiroplasma host adaptation was discussed. In addition, two *S. citri* strains differing notably in their ability to express adhesin ScARP2b and pyruvate dehydrogenase E1 component differed in their capacity to multiply in the two hosts, the plant and the leafhopper vector.

**Conclusions:**

This study provided us with a list of genes differentially expressed in the different hosts, leading to the identification of factors that are thought to be involved in the process of *S. citri* host adaptation. The identification of such factors is a key step for further understanding of *S. citri* pathogenesis. Moreover the present work highlights the high capacity of *S. citri* in tightly regulating the expression level of a large set of surface protein genes, despite the small size of its genome.

**Electronic supplementary material:**

The online version of this article (doi:10.1186/s12866-016-0666-y) contains supplementary material, which is available to authorized users.

## Background

*Spiroplasma citri* is the etiological agent of stubborn disease of citrus in the Mediterranean area and California [1] as well as horseradish brittle root disease in the United States [2]. It has a complex life cycle that involves multiplication in the insect vector and in the host plant, indicating that *S. citri* has the ability to adapt to two very different hosts. *S. citri* is transmitted from plant to plant in a persistent propagative manner by phloem sap-feeding insects of the order Hemiptera. Once ingested from the phloem vessels of an infected plant by the leafhopper vectors, *Circulifer tenellus* or *Circulifer haematoceps, S. citri* invades the entire insect. The circulative route of *S. citri* through its leafhopper vector is well established: spiroplasmas cross the insect gut wall, move into the hemolymph where they multiply, circulate, and invade most of the insect organs including the salivary glands, and are released in the main salivary duct leading to the stylet’s salivary canal. They are then introduced into the plant phloem along with salivary secretions during feeding [3–5]. In the host plant, *S. citri* multiplies in the phloem sieve elements and triggers severe symptoms. In the experimental host plant (periwinkle *Catharanthus roseus*), *S. citri* induces leaf yellowing, wilting and stunting [1]. The molecular mechanisms underlying the interaction between *S. citri* and its host plant remain largely unknown. Nonetheless, a perfect correlation between the ability of *S. citri* to use fructose and its ability to induce symptoms in the host plant was demonstrated [6].

In bacterial pathogens, many lipoproteins have been shown to play a key role in virulence-associated functions such as adhesion, invasion and colonization [7, 8]. In *S. citri*, surface proteins are suspected to recognize the insect gut and/or salivary glands epithelium, possibly participating in both adsorption and endocytotic events mediated by receptor ligand interactions [4]. The *S. citri* GII-3 genome (1,820 kbp) [9] encodes 645 membrane proteins including 68 putative lipoproteins, as predicted by the presence of a consensus lipobox in the first 28–30 amino-acids [10] and 577 transmembrane proteins [11]. The major lipoprotein at the cell surface of *S. citri* GII-3 is the protein named spiralin [12], which is required for efficient transmission of *S. citri* by its leafhopper vector [13] and, which was further shown to act *in vitro* and *in vivo* as a lectin able to bind glycoproteins of the vector insect [14, 15]. In addition, the surface lipoprotein Sc76 homolog to a solute-binding protein of an ABC transporter was also found to be involved as disruption of the gene dramatically reduced *S. citri* ability to be transmitted by *C. haematoceps* [16]. The *S. citri* GII-3 genome is also characterized by an abundant extrachromosomal DNA content, including seven plasmids, pSciA and pSci1 to pSci6, present as 10 to 14 copies per cell [17]. Plasmid genes also encode proteins associated with spiroplasma transmission. Recently, the role of the 8 surface adhesion-related proteins (ScARPs) encoded by plasmids pSci 1 to pSci 5 [17] has been studied. As compared to the wild-type strain GII-3, the *S. citri* mutant G/6 [18] and the non-insect-transmissible, strain 44 [19] both lacking pSci1 to 5, were affected in their ability to adhere and enter into the leafhopper cells [20, 21]. The role of ScARPs in adhesion and entry in leafhopper cells was clearly demonstrated for the ScARP3d, which possesses the whole set of domains found in ScARPs as well as the largest repeated domain [21]. Only a few membrane proteins have been investigated for their implication in transmission of *S. citri* by its vector insect, and the putative role of surface-exposed proteins in plant disease has not been studied. Moreover, despite surface proteins have been shown to be involved in transmission of *S. citri* by the leafhopper vector, very few is known about their regulation. Only one study dealt with gene regulation in *S. citri* [22]. In this work, the genes encoding the glucose and trehalose permeases were shown to be up-regulated in the presence of the respective sugars, reflecting the capacity of *S. citri* to adapt to environments with distinct carbohydrate contents [22].

Understanding the changes in membrane protein gene expression in response to different environmental conditions (plant and insect) is an important step in unraveling the possible functions of these genes and the transcriptional regulation mechanisms in *S. citri*. To investigate the molecular adaptation of *S. citri* in its different environments we compared the expression level of 28 putative lipoprotein genes including *spiralin* and *sc76* in *S. citri*-infected leafhoppers with those in *S. citri*-infected periwinkle plants. Expression profile of the *S. citri* ScARP genes was also assessed in the plant and in the leafhopper host. In addition, considering that spiroplasmas persisting in plants for a long period of time may lose the expression of genes necessary for insect host adaptation or may overexpress genes involved in plant long-term adaptation, insect-infected plants were compared to graft-inoculated plants for *S. citri* lipoprotein gene expression.

## Methods

### Spiroplasmas, plants and leafhoppers

The *S. citri* wild-type strain GII-3 (GII-3 wt) was first isolated from its leafhopper vector, *C. haematoceps*, in Morocco [23]. To avoid loss of transmissibility due to extensive in vitro passaging, the working strain was periodically subcloned and selected clones were submitted to experimental transmission to periwinkle plants via injection to its leafhopper vector *C. haematoceps* to confirm transmissibility and pathogenicity. After isolation from symptomatic plants, batches of *S. citri* cultures with low passage numbers (less than 5p) were stored at -25 °C until use.

*S. citri* G-GIP (this study) was isolated from *S. citri* GII-3 wt, graft-infected periwinkles (GIP) five months after grafting. In this strain the *scarp2b* mRNA transcript was not detected in the first passage culture (strain G-GIP1), but was readily detected after ten passages in the culture medium (strain G-GIP10). Spiroplasmas were grown at 32 °C in SP4 medium [24].

Intra-abdominal microinjection of *S. citri* into *C. haematoceps* leafhoppers and transmission to periwinkle host plant (*Catharanthus roseus*) were previously described [6, 25]. Leafhoppers were injected with low passage (7 to 10p) cultures of *S. citri* GII-3 and caged on healthy stock plants (*Matthiola incana*) for 2 weeks before being randomly divided in 2 groups. The first group was used directly for insect DNA extraction; the second group was transferred onto young periwinkle host plant (six-to eight–leaf stage, 10 insects per plant) for a period of 3 weeks (transmission period). Plants with symptoms were designated in our study as ‘leafhopper-infected periwinkles‘(LIP).

*S. citri* GII-3 was also maintained into periwinkle plants by successive graft inoculations without any insect transmission. In this case, inoculum sources were symptomatic branches of 1 year old plants originally infected with *S. citri* GII-3 via leafhopper transmission.

### DNA isolation and spiroplasma quantification by quantitative PCR

Total DNA from pure culture of *S. citri* was isolated by using the Wizard genomic DNA purification kit (Promega, Madison, Wis. USA). Five hundred milligrams of midribs collected on infected periwinkles (GIP or LIP) were ground in a plastic bag in a Homex 6 homogenizer (Bioreba AG, CH-4153 Reinach BL1, Switzerland). Total DNA was extracted using the CTAB (cetyl trimethyl ammonium bromide) method according to Murray and Thompson [26]. DNA from leafhoppers was also purified by the CTAB method. DNA preparations were kept at -20 ° C.

For quantitative real time PCR, the LightCycler® 480 SYBR Green I Master Mix (04887352001, Roche) was used. The SYBR Green reaction was performed in a 30 μl reaction mixture containing 1 X master mix, 0.15 mM of each primer, and 1 μg of total DNA preparation. The LightCycler® 480 System (Roche Diagnostics GmbH Mannheim, Germany) was used with the following program for DNA amplification: 95 °C for 15 min, 40 cycles each at 95 °C for 30 s, 67 °C for 30 s, 72 °C for 30 s, and a final extension at 72 °C for 10 min. Primers for quantitative amplification of *S. citri* DNA were designed from the spiralin gene [EMBL:Q2YHQ8]; the sequences of forward primer SQ1 and reverse primer SQ2 were 5′ ACAACGAAGGTACATCATTAACAAC 3′ and 5′ TTTGCTGGAGTAATTTGAACATAAAC 3′, respectively, and led to an amplicon of 80 bp.

For absolute quantification, plasmid pES3′ [27] containing the spiralin gene was used to construct the calibration curve and calculate the PCR reaction efficiencies. Knowing the number of plasmid molecules in 5 μl, tenfold serial dilutions of the plasmid DNA were prepared and used to generate the standard curve. To determine the theoretical sensitivity and the reliability of the qPCR, three repetitions of the assay were performed.

### RNA extraction from infected and uninfected hosts and cDNA synthesis

Total RNA from spiroplasma-infected plants, infected leafhoppers, and from spiroplasma cells in culture were isolated by using Trizol Reagent according to manufacturer’s guidelines (Invitrogen CA, USA). For periwinkle plants, 5 to 10 leaf midribs were ground in a mortar by freezing with liquid nitrogen and homogenized in 1 mL of Trizol reagent. Fresh leafhoppers (˜10) were ground directly in the Trizol Reagent. Total RNA from frozen *S. citri* cell pellets harvested by centrifugation during the exponential growth phase was extracted following the same procedure as above for the different hosts.

Subsequently, all RNA samples were treated with RNase-free RQ1 DNase (Promega, Madison, WI, USA) for 1.5 h at 37 °C to remove residual DNA, ethanol-precipitated, and finally dissolved in water following the protocol described by the supplier. DNase I treated RNA samples were tested in conventional PCR with primers SQ1-SQ2 without the RT step to confirm the absence of significant amounts of contaminating genomic DNA.

For each sample (spiroplasmas, healthy and infected hosts) 1 μg of DNA-free RNA was used for cDNA synthesis using Superscript Reverse Transcriptase III and random or specific primers according to manufacturer’s guidelines (Invitrogen, CA, USA).

### Quantitative real time reverse transcription PCR (RT-PCR)

Quantitative real time RT-PCR assays were performed on cDNA templates using the SYBR green chemistry detection system according to the manufacturer’s instructions (Roche Diagnostics GmbH Mannheim, Germany). In order to validate changes in transcript levels, identification of reference genes whose expression is independent of the environmental conditions is required. The genes selected to be tested as possible reference transcripts are listed in Table 1. Quantitative real time RT-PCR was performed using 5 μl of template in 1 X Light Cycler 480 SYBR Green Master Mix and 0.15 μM of each primer in a total volume of 25 μl. Primers used in the qRT-PCR assays for evaluating the expression of *S. citri* lipoproteins in leafhoppers and plants were listed in Table 2.

**Table 1:** List of selected genes to be tested as reference genes in the present study

Name	GenBank accession	Symbol	Function	Primer sequences (5′ to 3′) foward/reverse	Amplicon length
Putative chromosomal replication initiator protein dnaA	SPICI01B_001	*dnaA*	Replication	ATGAGTAAATCACGAGTTAG TCTTTGCCACCGAACTCTG	116
Dna gyrase subunit b protein	SPICI01B_003	*gyrB*	Topoisomerase	GGAGATTCTGCTGGTGGAAGTG TCTTTAATACCTGCTCCTAATGCG	167
Dna gyrase subunit a protein	SPICI01B_004	*gyrA*	Topoisomerase	TTCGCCAAACAGGGAAAGTAG CTCCAGTAGCATCATTAGCAATTC	195
Dna-directed rna polymerase beta chain protein	SPICI01B_073	*rpoB*	Transcription	TGTGCCATTAGTGCGTCAAG CATCTTCTGATACTAAGCGTTCTG	179
Hypothetical chromosome replication initiation and membrane attachment protein	SPICI03_040	*dnaB*	Replication	AATTACCAATTTCCGCAATTGC TTGTTTGTCTTCTTGATTATTAAC	131
50s ribosomal protein L3	SPICI03_102	*rplC*	ribosomal protein L3	AATGCCTGGACATATGGGAAC GCATCAACAACTACAACTGG	252
Spiralin lipoprotein	SPICI04_139	*spi*	Lipoprotein	ACAACGAAGGTACATCATTAACAAC TTTGCTGGAGTAATTTGAACATAAAC	80
Pyruvate kinase protein	SPICI04_141	*pyK*	Glycolysis	GGGAATTATTAAAAACAATTTC TTGCCACTTCACAAATTGC	171
Fibril protein	SPICI12_006	*fib*	Cytoskeleton Structure	TAAGCATGATACAGGAGATACAAC TGCCCATATTTATCAACCATTTCC	246
Cell shape-determining protein mreb1	SPICI13_009	*mreB1*	Cell morphogenesis	AGGAACAACAGACATTGCGG TCTCTAGCCCATATTGAGAAC	125
16S rRNA	ND	*16S*	30S ribosome subunit RNA component	CAAATCCTGGAGCTCAACTC GCGTAGACTACTAGGGTATC	204

**Table 2:** List of primers used to study lipoprotein genes expression in *Spiroplasma citri* GII-3

Name	Primers sequences (5′ → 3′)	Gene product	Amplicon length (bp)	Annealing temperature (°C)	Efficiency (%)
pSci4_02	GGCAATGACTTCAAGTTCGTG and TGTTTTCTCTTACTGTTGATGG	Hypothetical lipoprotein	221	52	99.9
pSci4_06	ATCAGTTAACAATGCTTCTGAG and TATCAGGCCTATCTTTACTATC	Hypothetical lipoprotein	334	52	91.0
pSci6_18	AGTGTTTCGCTCGGTTCTAG and GCATTTGCTTCACCAGATTTC	Truncated adhesion-related protein	173	60	93.9
SPICI01A_047	GATGTACGAATTCGCCAA and TCGATTCGTTGTTTTGCTTC	Hypothetical lipoprotein	563	52	102.8
SPICI02_046	TGCAACAACCAAGTTTCCAAG and TAGCAAGAACCGTATTTCCATG	Hypothetical lipoprotein	288	60	95.9
SPICI03_030	AGTAACATCACCAACCTTATTG and ATCGGTTGCTATTGTACCATC	Hypothetical lipoprotein	219	60	103.0
SPICI03_098	GTTTACAGGGAGGGCGAATG and TTGCAAGATAACGTGCTGATTG	Hypothetical lipoprotein	573	60	100.2
SPICI03_180	TTGGGAAAAGGCAGTTGGTAG and CTGTTCGCCCAATATTAGGTC	Hypothetical lipoprotein	659	60	100.0
SPICI03_317	GAAATAGTTTTGATAATGAGTTTAG and GCAGTGTTAAACATTACAAAATC	Hypothetical lipoprotein	184	52	108.4
SPICI04_017	CACCAGTTTCAAACCCAAC and AATTACTGCTGATTCATTAGG	Hypothetical lipoprotein	86	60	99.7
SPICI04_108	ACTTCGGCTTCTATTACTTCAG and CCTGGATCAAGATCAACAGC	Hypothetical lipoprotein	157	60	100.5
SPICI04_139	ACAACGAAGGTACATCATTAACAAC and TTTGCTGGAGTAATTTGAACATAAAC	Spiralin	80	60	100.8
SPICI05_014	CCGGTATAACCTTTTGTCAC and AATTAGTTCAACGCTTTGAG	Hypothetical lipoprotein	138	60	96.1
SPICI06_025	CTAATACACAACAACCGCCC and CTTTACACCAGATGTATCGTG	Hypothetical lipoprotein	161	60	103.1
SPICI07_030	CTTCCCGTACTTACTAACG and ATACTAAAGATTTGGGAGGC	Hypothetical lipoprotein	160	52	108.8
SPICI09_027	TTGCCCGCTAATATCTTTTG and TGATTTATGAAATATGATGGTC	Hypothetical lipoprotein	153	52	108.8
SPICI10_054	CATCCGGATTTGCAATCAAACC and CAGCGCTTGTCAATTACTGC	Hypothetical lipoprotein	505	60	97.8
SPICI10_055	GGTGACGAAGGAATTGATGC and CCTGCGCTCATTGTAACATC	Hypothetical lipoprotein	206	60	96.1
SPICI11_003	GTGCAATTAAAAGTAGG and GTGCAATTAAAAGTAGG	Sc76	157	52	104.6
SPICI12_020	TGCTACTGTTGTTAGTTGTGC and CTCAATTGCAATTTCACCACG	Hypothetical lipoprotein	200	60	96.3
SPICI12_021	TGATGCACCACTGAAAATTGG and CGGCAACATCAGGATTATGG	OppA	536	60	98.2
SPICI12_028	ACGGTTATTAACACTTTTTAGTG and TCCAAGATCTTGATGACCTTC	Hypothetical lipoprotein	126	60	95.6
SPICI13_014	AACCAATTGAACCACCAGAAG and CACAATCATAGACAATTGCTTG	Hypothetical lipoprotein	228	60	98.4
SPICI16_011	GTCAATGCCACCGTTTAATGC and AGCACCAGGAATGAAAACAGC	Hypothetical lipoprotein	535	52	90.6
SPICI20_004	GAATTATGATGAGGAGAC and AAGTTAAAGTAATTCCTGC	Hypothetical lipoprotein	191	60	93.3
SPICI20_057	TTGATGAATCGCTTTCCTATTG and CTTGTGCCATTATTGTATAACC	Hypothetical lipoprotein	360	60	95.5
SPICI20_065	GTGAAGGCACAGTTACTCC and GCTGAGCCAGAACTTGAAC	Hypothetical lipoprotein	562	60	100.3
SPICI20_066	TTAAGCGCTATGGTAGTGGC and ATACCTGGTGTTGCTGTGTC	Hypothetical lipoprotein	445	60	91.8

The relative quantification method (ΔΔC_T_) [28] was used to evaluate quantitative variation between the hosts (plant or leafhopper) and the culture arbitrary designated as “calibrator” in our study. For the calibrator sample, the average Ct value of the reference gene was subtracted from the Ct value of the target gene under investigation to give ΔC_T calibrator_. The ΔC_T_ values for plants or insects were calculated using the same procedure. The ΔΔC_T_ value calculated for each host was obtained by subtracting the respective ΔC_T_ of the target gene in the calibrator sample from those of the target gene in the host. The data were analyzed using the following equation [28].

Experiments were carried out on three independent biological replicates, each consisting of three replicate reactions. A significant change in ΔΔC_T_ value in host *versus* axenic medium was considered if ΔΔC_T_ value was superior to 1. To identify genes for which expression was significantly different in various hosts, statistical analyses were performed using Student’s t test (*P* < 0.05).

### Bi-dimensional gel electrophoresis and nLC-MS/MS analysis

Spiroplasma proteins from 150 mL culture were prepared as previously described [29]. Proteins (300 μg) solubilized in 1–5 mL of a rehydration solution containing 7 M urea, 2 M thiourea, 4 % (w/v) CHAPS, 2 % Triton X-100, 10 mM DTT and 2 % (v/v) Ampholine pH 3–10 were submitted to 2-D gel electrophoresis as described before [29]. Gels intended for LC-MS/MS analysis were stained using Coomassie brilliant blue [30]. For the nLC-MS/MS analysis, the gel spot (1 × 1 mm) present in the 2D-gel obtained for *S. citri* GII-3 wt expressing the *scarp2b* gene was excised then treated with destaining solution consisting of 25 mM ammonium bicarbonate and 50 % acetonitrile (ACN). The gel piece was rinsed twice in ultrapure water and shrunk in ACN for 10 min. After ACN removal, the gel piece was dried at room temperature, covered with trypsin solution (10 ng/μL in 40 mM NH_4_HCO_3_ and 10 % ACN), rehydrated at 4 °C for 10 min, and finally incubated overnight at 37 °C. The gel piece was then incubated for 15 min in 40 mM NH_4_HCO_3_ and 10 % ACN at room temperature with rotary shaking. The supernatant was collected, and an H_2_O/ACN/HCOOH (47.5:47.5:5) extraction solution was added onto the gel piece for 15 min. The extraction step was repeated twice. Supernatants were pooled and concentrated in a vacuum centrifuge to a final volume of 25 μL. Digests were finally acidified by addition of 2.4 μL of formic acid (5 %, v/v) and stored at -20 °C. The peptide mixture was analyzed on a Ultimate 3000 nanoLC system (Dionex, Amsterdam, The Netherlands) coupled to an Electrospray LTQ-Orbitrap XL mass spectrometer (Thermo Fisher Scientific, San Jose, CA). The conditions of peptide separation and data acquisition were identical to those described in [31]. Data were searched by SEQUEST through Proteome Discoverer 1.4 (Thermo Fisher Scientific Inc.) against a custom made *S. citri* GII-3 database. Spectra from peptides higher than 5000 Da or lower than 350 Da were rejected. The search parameters were as follows: mass accuracy of the monoisotopic peptide precursor was set to 10 ppm and peptide fragments tolerance was set at 0.6 Da. Only b- and y-ions were considered for mass calculation. Oxidation of methionines (+16 Da) and deamidation of asparagine and glutamine (+1 Da) were considered as variable modifications and two missed trypsin cleavages were allowed. Only high-confidence matches corresponding to false positive rate of 1 % at peptide level were considered.

## Results and discussion

### Quantification of spiroplasmas in periwinkle plants and insects

From the standard curve constructed with serial dilutions of the plasmid pES3′, real-time PCR assay was used to accurately quantify the spiroplasma cells in plant and insect extracts used in this study. One ng of pES3′ contains 10^8^ molecules of plasmid, each containing one copy of the spiralin gene. Because this gene is present in a single copy on the spiroplasma chromosome, 1 ng of pES3′ corresponds to 10^8^ spiroplasmas. The number of spiroplasma cells in 1 μg of DNA extracted either from periwinkle plants infected by insects (LIP) or grafted periwinkle plants (GIP) fresh midribs or from infected insects was similar and corresponded to 1.5 ± 0.5 × 10^5^, 1.5 ± 0.7 × 10^5^, and 1.8 ± 0.6 × 10^5^, respectively. The average number of spiroplasmas in 1 g of fresh midribs from LIP reached 2.4 ± 0.8 × 10^7^; from GIP the average value was 3.3 ± 1.7 × 10^7^. The number of spiroplasmas in leafhoppers was equivalent to 6.4 ± 2.4 × 10^6^ spiroplasma cells per insect.

### Selection of reference genes for transcript quantification in plants and leafhoppers

The overall cycle threshold values C_t_ for the 11 genes selected as candidates for normalization of transcript level determination (Table 1) in plants and leafhoppers were distributed from 15 to 30. Control reactions without *S. citri* template, performed with cDNAs from healthy plants and insects, remained below the threshold for genes *16S, spi, fib*, *pyk*, *rplC*. Weak unspecific signals detected with *rpoB, gyrA* and *B, dnaA* and *B*, and *mreB* in the no template controls excluded these genes from the study.

For four candidate genes *fib*, *pyk*, *rplC*, and *spiralin,* transcript level in the 2 hosts was analyzed by absolute quantification. For each gene, a standard curve prepared with known concentrations of the same gene previously cloned in a plasmid gave regression lines with an average slope value of -3.542 and an average error value of 0.04. All PCRs displayed an efficiency ranging from 96 to 99 %. Such efficiency is considered acceptable and this relatively high-efficiency value results in a better sensitivity at low target concentrations.

**Fig. 1:**
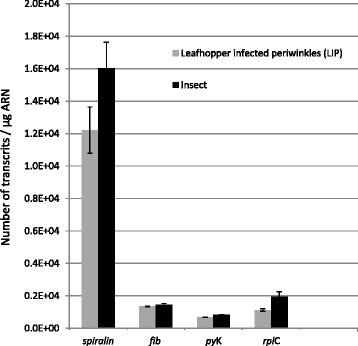
Absolute quantification of 4 candidate reference genes (*spiralin*, *fib*, *pyk* and *rplC*) in leafhopper-infected plants (LIP) and in insects. Grey bars indicate the number of transcripts detected in LIP and the black bars the transcript level in insects. Bars correspond to the standard deviation obtained with three independent replicates

As shown on Fig. 1, expression of *spiralin* and *rpl*C varied according to the hosts and were expressed at higher levels in leafhoppers than in plants. Also *spiralin* transcripts were 10 times more abundant in both hosts than the other gene expression products. Thus these 2 genes cannot be used as internal controls. Comparison of the transcript levels of *fib* and *pyk* in infected leafhoppers and plants (Fig. 1) showed that each of these genes was equally expressed in both environments. Transcript levels of *fib* and *pyk* in leafhoppers and plants were then compared to those in the culture medium. The number of transcripts per spiroplasma was not significantly different in leafhoppers, in Lip and in the culture medium (0.1 ± 0.02 for *fib* and 0.07 ± 0.001 for *pyk*). Considering that *fib* and *pyk* were equally transcribed in the three environments these genes could be used as internal controls. Given that the fibril protein, but not Pyk, is specific to spiroplasmas [32], *fib* was used as the reference gene in further experiments.

### Lipoprotein genes expression profiles in the different environments

To investigate the molecular adaptation of *S. citri* in plants and leafhoppers, we examined the expression of spiroplasma lipoprotein genes. Among the 68 genes predicted to encode lipoproteins, genes with redundant sequences (of viral origin), pseudogenes (with the exception of pSci6_18), as well as genes, for which no satisfactory amplification primers could be identified were removed from the study. Among the 28 selected genes, 3 were carried by plasmids pSci4 and pSci6, and 25 others, including genes *spiralin* (SPICI04_139) and *sc76* (SPICI11_003) were carried by the chromosome (Table 2). Evaluation of mRNA expression profiles in both environments was conducted in leafhoppers, and in the 2 types of periwinkle plants LIP and GIP (see section Methods). The relative gene expression level was calculated as described in Methods where the transcript level in SP4 medium of the target and reference (*fib*) genes were chosen as calibrators. All PCRs displayed an efficiency ranging from 90.6 to 108.8 %.

**Fig. 2:**
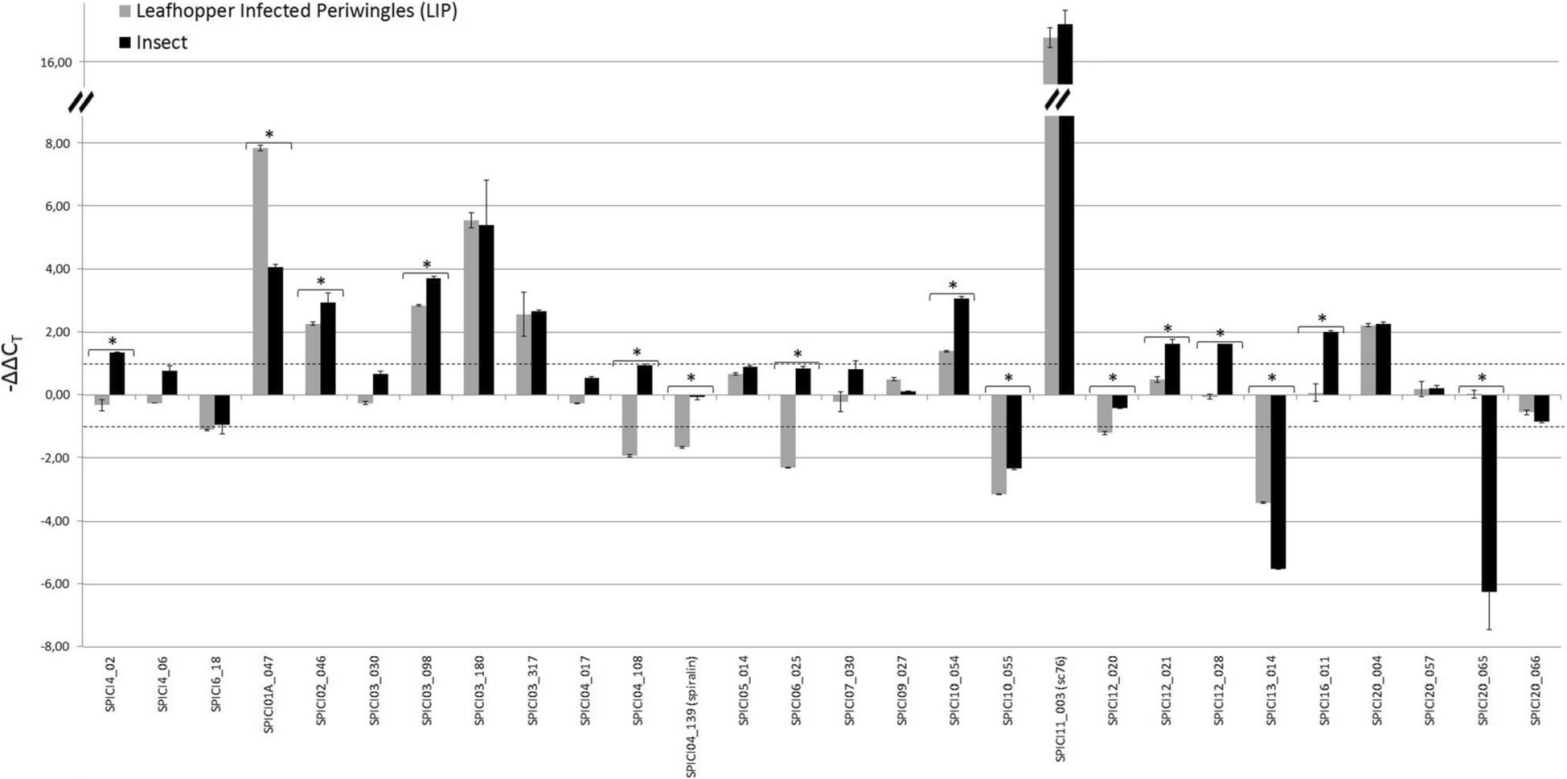
Comparison of the relative expression levels of *S. citri* lipoprotein genes (-ΔΔC_T_) in leafhopper-infected plants (LIP) and in insects. The -ΔΔC_T_ value calculated for each host was obtained by subtracting the respective ΔC_T_ of the target gene in the calibrator sample corresponding to axenic medium from those of the target gene in the host. Positive -ΔΔC_T_ value indicate an up-regulation of the target gene, while a negative value indicates its down-regulation. Experiments were carried out on three independent biological replicates, each consisting of three replicate reactions. A change in │ΔΔC_T_│ in host *versus* axenic medium was considered as significant if superior to 1 (either above (up-regulated genes) or below (down-regulated) the dashed lines). Asterisks indicate genes, for which the expression level is significantly different in insects and in LIP as determined using the Student’s t test (*P* < 0.05, │ΔΔC_T_│ > 1 in at least one host). Grey bars indicate -ΔΔC_T_ measured in LIP and the black bars the -ΔΔC_T_ in infected insects

The calculated -ΔΔCt values for the 28 tested genes are shown in Fig. 2 and in Additional file 1: Table S1. A positive –ΔΔCt value indicates an up-regulation of the gene’s expression whereas a negative –ΔΔCt indicates down-regulation as normalized to the *fib* reference gene.

#### Lipoprotein gene expression profiling in plant LIP and leafhopper hosts

*Lipoprotein gene expression profiles in infected insects versus spiroplasmas cultured in SP4* (black bars, Fig. 2). During cultivation in SP4 medium all 28 *S. citri* lipoprotein genes were expressed. Expression of fifteen of them significantly changed once the spiroplasmas were introduced in insects, indicating the strong transcriptional response of *S. citri* to environmental changes. Among these genes, 12 (including SPICI11_003 (*sc76*) and SPICI12 _021 (*oppA*)) were up-regulated, while 3 were down-regulated in insects. These were operationally defined as “insect-up-regulated” and “insect-down-regulated”, respectively.

*Lipoprotein gene expression profiles in spiroplasmas from leafhopper inoculated plants (LIP) versus those in spiroplasmas grown in culture* (grey bars, Fig. 2)*.* The pattern of lipoprotein gene expression was found to be clearly different in LIP. In the host plant, 8 lipoprotein genes were up-regulated and 7 genes (including *spiralin*) were down-regulated.

*Lipoprotein gene expression profiles in spiroplasmas from leafhopper inoculated plants (LIP) versus those in spiroplasmas from leafhopper bodies* (grey bars versus black bars, Fig. 2). Eight genes were up-regulated in both plant and insect hosts, whereas 2 were significantly down-regulated in both hosts. Fifteen genes (for which a │ΔΔC_T_│ > 1 was obtained for at least one host compared to SP4) were differentially expressed in insects and in plants (indicated by an asterisk on Fig. 2). For most of them (12/15), the transcript level was higher in insects than in plants. Four genes (SPICI04_108, *spiralin*, SPICI06_025 (prophage element), SPICI12_020) were down-regulated in plants but not in the leafhopper. Genes pSci4_02, SPICI12_021, SPICI12_028, SPICI16_011 were overexpressed in insects but neither up- nor down-regulated in LIP. Expression of SPICI20_065 transcripts was repressed only in insects (no activation or repression in infected LIP) compared to SP4. Six genes followed the same type of regulation in both hosts but showed significantly different transcript levels in LIP and in insects (SPICI01A_047, more strongly up-regulated in plants; SPICI02_046, SPICI03_098, SPICI10_054 more strongly up-regulated in insects; SPICI13_014, more strongly down-regulated in insects; SPICI10_055 more strongly down-regulated in plants).

To summarize, during *in vitro* cultivation of *S. citri* in SP4 medium, the 28 lipoprotein genes tested were expressed. Once the spiroplasmas were introduced into the leafhopper host, 12 of the lipoprotein genes were up-regulated. Among these genes that are up-regulated, and thus putatively involved in adaptation of the bacteria to its insect host, the majority encode hypothetical lipoproteins with no assigned function (Table 1). However, two of them (SPICI11_003 (*sc76*) and SPICI12 _021 (*oppA*)) are noticeable, as they encode proteins sharing sequence identity with substrate binding units of two distinct ABC transporters. The superfamily of ABC transporters plays an important role in the export of proteins and polysaccharides and in the import of sugars, inorganic ions, and oligopeptides [33]. SPICI11_003 (*sc76*) was found to be up-regulated in both insects and plants, suggesting that this gene may be required in both hosts. Sc76 is a solute binding protein of a sugar ABC transporter, for which the sugar specificity has not yet been identified [16]. Sc76 could play a role in *S. citri* GII-3 growth, as the *S. citri* mutant G76 having a truncated *sc76* gene multiplies to low titers in plants and leafhopper salivary glands compared to GII-3 wt [16]. In plants sucrose is the most abundant sugar and the spiroplasma’s preferred sugar is fructose [34], while the main sugar in the insect hemolymph and salivary glands is trehalose [35, 36]. The fact that, in plants and insects, the transcription of *sc76* was up-regulated at a similar level suggests that expression of *sc76* might be regulated by a sugar present in both hosts. Even if glucose, which is abundant in SP4 medium can be easily excluded, it remains to be investigated which sugar might be transported through the Sc76-containing ABC transport system. Interestingly, *S. citri* pathogenicity was severely impaired in fructose operon mutants [6], and transcription of the genes coding for the phosphoenolpyruvate transferase systems (PTS) responsible for glucose and fructose import into the spiroplasma cell were stimulated by the respective sugar [22, 37]. The present work provides further evidence of the crucial role of sugar metabolism in spiroplasma pathogenicity, and suggests that the sugar transported via Sc76 could participate in *S. citri* ‘s adaptive capacity to multiply in distinct hosts.

The lipoprotein SPICI12_021 shares identity with the substrate binding unit OppA of the oligopeptide permease. Over the past 10 years OppA has been characterized as a multifunctional lipoprotein in mollicutes. As for an example, OppA was not only involved in oligopeptide import into the cytoplasm, but also in cytadherence to and invasion of epithelial surfaces of the human urogenital tract by *M. hominis* [38]. In *M. pneumoniae* a lipoprotein gene (*mpn456*) having with homology to a gene encoding a predicted oligopeptide ABC transport system was also up-regulated in response to adhesion to a human cell line [39]. More generally, in several bacteria other than mollicutes, such as *Mycobacterium tuberculosis*, solute binding proteins of ABC transporters are lipoproteins that play a role in bacterial growth and contribute to virulence [8]. As in the human mycoplasmas *M. hominis* and *M. pneumoniae*, the protein SPICI 12_021 could play a key role in the spiroplasmas’ interactions with leafhopper cells, which are crucial steps for transmission of *S. citri* by its vector insect.

The *spiralin* gene was shown to be strikingly down-regulated in plant (LIP) whilst it was abundantly expressed in insects and in culture. Spiralin is the most abundant lipoprotein of *S. citri* membrane, and covers the entire spiroplasma cell surface [12, 40]. This lipoprotein was designated as a lectin interacting with insect glycoproteins [14, 15] and was required for adhesion and entry of *S. citri* into insect cells [15]. Thus it could be hypothesized that, during transmission of *S. citri* to plant hosts, over-expression of spiralin within the leafhopper vector would occur to enable adhesion and internalization of spiroplasmas into midgut and salivary glands cells.

These results revealed infection regulatory programs common to both hosts as well as genes submitted to insect- or plant- specific regulation, indicating a fine-tuned regulation of several lipoprotein genes depending on the *S. citri* environment, despite the reduced genome size of this bacterium [9]. Guell et al. [41] have analyzed large transcriptomic data sets obtained with *M. pneumoniae* cultivated under a broad range of conditions and submitted to diverse stresses. Their study highlighted the unanticipated, high transcriptome complexity in mollicutes. Considering that *M. pneumoniae* possesses one of the smallest genomes among mollicutes, it is plausible that a high level of transcriptional regulation also occurs in other mollicutes upon environmental changes. Nevertheless, most transcriptional variations that occur in mollicutes upon environmental changes have been recorded *in vitro*, and there are only a few *in vivo* studies [39, 42, 43]. Unlike mycoplasmas, *S. citri* invades two very different hosts and our data demonstrate differential expression of genes encoding membrane-anchored proteins in plants and in insects. This study provided us with a list of lipoprotein genes putatively involved in *S. citri* adaptation to its hosts and possibly underlying virulence and/or host specialization. Genes pSci4_02, SPICI10_054, SPICI12_021, SPICI12_028, and SPICI16_011, which, like *spiralin*, *sc76,* and *oppA*, are overexpressed in the leafhopper, and SPICI04_108, SPICI06_025 (prophage element), and SPICI12_020, which are down-regulated in plants are therefore good candidates for being involved in the adaptation of *S. citri* to its insect host. On the contrary, genes that are strongly up-regulated in LIP (such as SPICI01A_047) or are less repressed in plants than in insects (SPICI13_014, SPICI20_065) are expected to encode proteins involved in adaptation to the host plant.

#### Comparison of lipoprotein gene expression in LIP and GIP plants

To analyze the putative adaptive response of *S. citri* during long-term plant infection, lipoprotein genes’ expression in leafhopper-infected periwinkles LIP (recent infection) was compared to those in graft-infected periwinkles GIP, in which *S. citri* GII-3 wt was inoculated through grafting 5 months earlier (old infection). In both cases (graft- and insect-inoculation) the infected plants share similar symptoms suggesting they share similar physiological responses to *S. citri* infection.

**Fig. 3:**
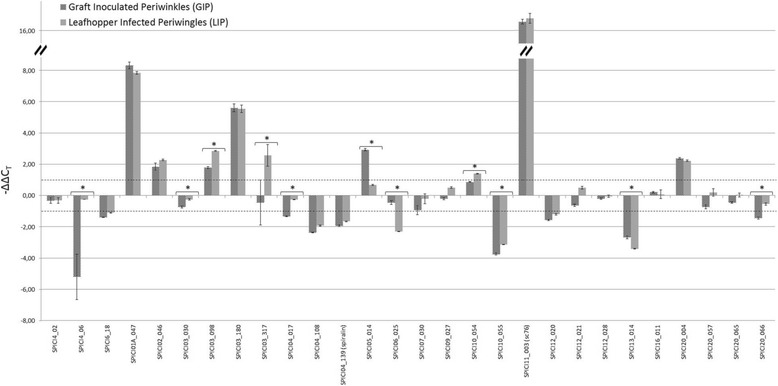
Comparison of the relative lipoprotein genes expression levels (-ΔΔC_T_) in graft-inoculated plants (GIP) and in leafhopper-infected plants (LIP). The -ΔΔC_T_ value calculated for each host was obtained by subtracting the respective ΔC_T_ of the target gene in the calibrator sample corresponding to axenic medium from those of the target gene in the host. Positive -ΔΔC_T_ values indicate an up-regulation of the target gene, while a negative value indicates its down-regulation. Experiments were carried out on three independent biological replicates, each consisting of three replicate reactions. A change in │ΔΔC_T_│ in host *versus* axenic medium was considered as significant if superior to 1 (either above (up-regulated genes) or below (down-regulated) the dashed lines). Asterisks indicate genes, for which the expression level is significantly different in GIP and in LIP as determined using the Student’s t test (*P* < 0.05, │ΔΔC_T_│ > 1 in at least one host). Dashed bars indicate -ΔΔC_T_ measured in GIP and light grey bars indicate the -ΔΔC_T_ in LIP

Six spiroplasma lipoprotein genes were up-regulated in GIP and LIP infected hosts (Fig. 3 and Additional file 1: Table S1). Among them, SPICI01A_047 was less expressed in insects. The up-regulation of this gene could be involved in the protection of *S. citri* from plant defence or in the successful colonization of host plant by the spiroplasma. Three genes (pSci6_18, SPICI04_108, SPICI04_139 (*spi*)) were down-regulated in both GIP and LIP hosts and to a similar extent, suggesting that these genes are likely not to contribute to the adaptation of *S. citri* to the plant host.

For one gene SPICI 05_014 overexpression level was significant in GIP but not in LIP, in which the transcript level was similar to that measured in the leafhopper and in the SP4 culture medium. Instead, the plasmid gene pSci4_06, and the chromosomal genes SPICI04_017, SPICI20_066 were transcribed at a very low level in GIP, while their expression did not significantly differ in LIP and in leafhoppers. The changes of expression of these genes in GIP compared to LIP could be due either to the age of the host plant, to the duration of infection, or to the mode of inoculation (graft *vs* insect-infection), three variables differing in LIP and GIP infected-plant models. Due to differences between old and young plants in the efflux of essential micronutrients such as sugar and amino acids in the phloem sap, changes in gene expression between old-grafted (GIP) and young (LIP) periwinkle plants could reflect an adaptation to the old plant environment. Variation of gene expression in GIP compared to LIP could also be observed for genes having a role during the early stage of infection but not for spiroplasma persistence, when *S. citri* is well adapted to the host plant (GIP). Finally, in the case of GIP, the lack of an interim period of habitation in insects might be responsible for down-regulation of spiroplasma genes that are non-vital for survival in plants but are involved in adaptation to insects. Indeed the lack of exposure to the selective pressure exerted in the insect host is likely to alter the expression of such genes.

### Transcriptional analysis of *scarps* in *S. citri*

Eight *S. citri* adhesion-related proteins (ScARPs) are encoded by plasmids pSci1 to 5 whose presence has been associated with the ability of *S. citri* to be transmitted by its leafhopper vector [20, 21]. To determine whether *scarp* genes, similarly to several lipoprotein genes, were regulated by environmental conditions, their expression levels in SP4, in infected insects, GIP and LIP were compared. To avoid misinterpretation due to variable copy number of the different plasmids, the *scarp* genes *scarp*5a and *scarp*2b, carried by the same plasmid (pSci5) [17], were chosen for comparison.

**Fig. 4:**
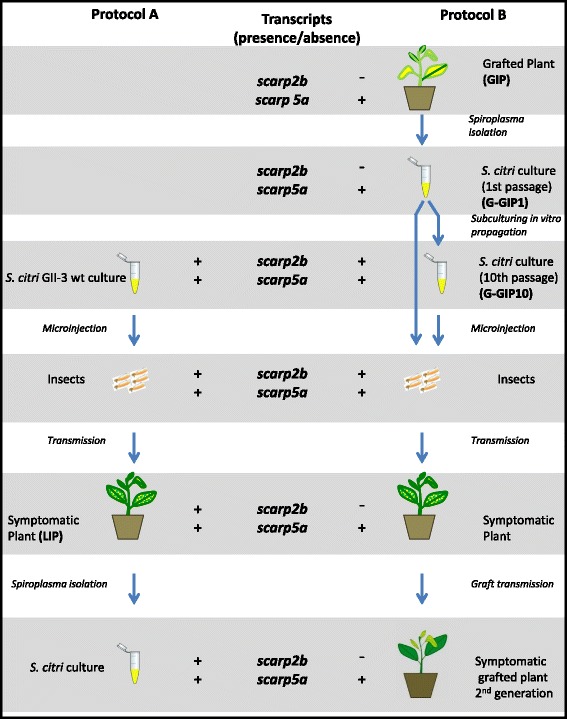
Schematic representation of the protocols used for *scarp* expression studies in the different hosts and expression of scarps under these conditions. Positive and negative detection (see Methods for details) of *scarp2b* and *scarp5a* transcripts in the different hosts are noted ‘-‘ or ‘+’, respectively. Protocol A (left column): A culture of *S. citri* in axenic medium was injected in insects, which then were fed on young periwinkles that became symptomatic within 3 weeks (leafhopper-infected plants LIP). *S. citri* extracted from LIP were then cultivated in axenic medium. Protocol B (right column): 5 months after inoculation, graft inoculated plants exhibiting symptoms (GIP) served as source of *S. citri* cultivated in axenic medium. After one passage, *scarp2b* transcripts were undetectable. After 10 passages, *scarp2b* transcripts could be detected, and spiroplasmas were microinjected to insects. The insects were fed on young periwinkles. Symptomatic periwinkles were used to graft a new batch of periwinkle plants, which developed symptoms (grafted plants second generation)

In *S. citri* GII-3 wt culture the levels of *scarp 2b* and *5a* transcripts per μg of RNA were similar, respectively 10 ± 1 × 10^6^ and 15 ± 1.25 × 10^6^. In infected leafhoppers, 12 days after injection the number of spiroplasma cells was 6 × 10^6^ per insect and the *scarp 2b* and *5a* transcript amounts were the same (35 ± 0.4 × 10^3^ for 1 μg of total infected leafhopper RNA). Plants infected by *S. citri* through leafhopper transmission (LIP) developed symptoms within 3 weeks and spiroplasmas reached a titer of 10^6^-10^7^ per g of fresh midribs. *Scarp2b* and *scarp5a* were equally transcribed to 10 ± 1 × 10^4^ transcripts per μg of total infected plant RNA. These results indicated that expression of *scarp 2b* and *5a* were similar in the culture medium, the infected leafhoppers and in LIP (Fig. 4, protocol A). On the contrary, *scarp2b* transcript was not detected in GIP (Fig. 4, protocol B), whereas expression level of *scarp5a* was identical to that detected in infected LIP. PCR amplifications and sequencing the *scarp2b* and *5a* coding sequences and the non-coding regions upstream of *scarp* genes revealed that in both LIP and GIP the sequences were 100 % identical to those of GII-3 wt (data not shown), indicating that no sequence deletions had occurred in these regions.

There are no existing mechanisms that explain how a given gene such as *scarp2b* can be down regulated in old grafted plants. To further investigate the influence of a long period of multiplication in plants (GIP) on *S. citri* adaptation, spiroplasmas were isolated from GIP, subcultured for 10 passages, and injected into leafhoppers (Fig. 4, protocol B). The expression levels of *scarp2b* were compared in the different environmental conditions explained in Fig. 4 (protocol B *vs* A).

In the first passage of the *S. citri* culture obtained from GIP, the *scarp2b* mRNA transcript was not detected. However after further passaging in the SP4 culture medium, the *scarp2b* transcript was detected and reached a 10^7^ transcripts/μg of RNA at the 10^th^ passage. Interestingly, in the leafhoppers injected with this 10 passages culture as well as in those injected with the early passage culture from GIP (which we named G-GIP1), expression level of *scarp2b* was similar to that obtained with leafhoppers injected with *S. citri* GII-3 wt. Unexpectedly, in periwinkle plants infected by these insects, expression of *scarp2b* was not detected whereas that of *scarp5a* was equivalent to that obtained in plants infected by GII-3 wt. Furthermore, in a periwinkle plant graft inoculated with a shoot coming from the newly infected plant (protocol B), *scarp2b* transcript was still undetectable. The fact that the *scarp2b* transcript was detected in leafhopper-infected periwinkles (LIP) (Fig. 4, protocol A) but not in plants infected or graft-inoculated by the *S. citri* originally isolated from GIP (Fig. 4, all symptomatic plants obtained through protocol B) strongly suggested a difference between the GIP-isolated spiroplasmas and *S. citri* GII-3 wt (used in protocol A). Therefore we investigated whether these results could be explained by a phenotypic (on/off expression of *scarp2b*) heterogeneity in the bacterial population present in GIP.

#### Spiroplasma mixture in grafted periwinkles

To challenge the hypothesis that GIP may contain a mixed bacterial population, spiroplasmas from GIP were directly plated on solid SP4 medium. After 10 days, spiroplasmas from 18 colonies were cultivated in liquid medium (only one passage) and submitted to qRT-PCR for detecting RNA transcription of *scarp2b*. Out of 18 spiroplasma cultures tested, 5 expressed the *scarp2b* RNA at levels similar to those obtained for GII-3 wt (10^7^/μg RNA) while the other 13 did not. Given that *scarp2b* transcripts could not be detected in GIP, such a high proportion of *scarp2b*-expressing colonies was unexpected. Following the hypothesis of a mixed population in GIP, *scarp2b*-expressing spiroplasmas could overtake the population that does not express *scarp2b* in the SP4 culture medium. Following this assumption the proportion of *scarp2b*-expressing colonies would provide an overestimation of *scarp2b*-expressing cells in GIP. This assumption is consistent with the finding that the *scarp2b* transcript level continuously increased during passaging in the culture medium.

One spiroplasma clone that expressed *scarp2b* and one that did not were separately injected into leafhoppers to investigate transmission to periwinkle plants. Ten days after transmission, all plants developed symptoms. In these plants, however, the scarp2b transcript was detected or not according to the initial spiroplasma inoculum. Thus, the phenotypic differences, in particular regarding *scarp2b* expression, between the *S. citri* clones are stable in plants and do not modify the pathogenicity to plants. These results confirmed the hypothesis that two phenotypes of *S. citri* co-existed in the original GIP. In these plants, the number of spiroplasma cells expressing *scarp2b* may fall below a detectable level whereas spiroplasmas lacking *scarp2b* expression efficiently multiplied. Other environments such as SP4 medium and leafhoppers probably constitute a better environment for propagation of *scarp2b* expressing spiroplasmas. The nature of the environmental selective pressure encountered by spiroplasmas differs in plants and in insects. This change may be responsible for the differential multiplication of the two phenotypic variants depending on the host. Taken together, our data suggest that this *scarp* gene may not be essential in plants and in insects (the *scarp2b* non-expresssing strain was transmissible), and argue in favor of the functional redundancy of *scarp* genes.

**Fig. 5:**
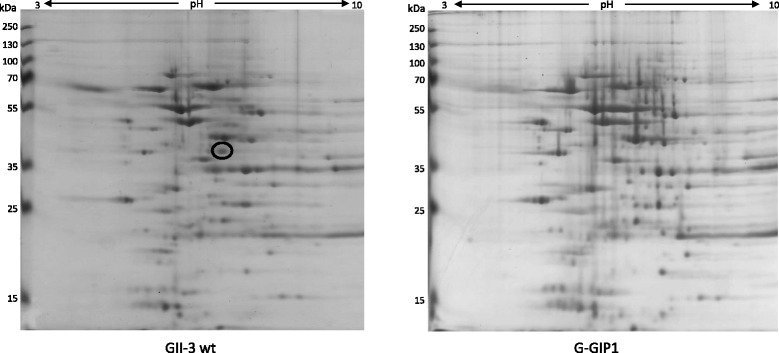
Bidimensional gel electrophoresis of total extract proteins of *S. citri* GII-3 wt and *S. citri* strain deficient in *scarp2b* expression *S. citri* G-GIP1. Circled spot was further analyzed by LC-MS/MS (see text for details)

Protein extracts from *S. citri* GII-3 wt and *S. citri* G-GIP1, which did not express *scarp2b*, were separated by 2D-electrophoresis to determine whether lack of *scarp2b* expression was associated with major changes in soluble protein expression profiles (Fig. 5). Two-dimensional electrophoresis patterns of the two strains were very similar for relative intensities of the protein spots, suggesting that these strains did not strikingly differ in their soluble protein expression profiles. However, one spot was clearly present in GII-3 wt strain and absent in G-Scarp-2b^-^. Due to the lack of their expression in spiroplasmas well-adapted to plants, the proteins present in this spot were considered as good candidates for being involved in adaptation of *S. citri* to the insect. *LC*-*MS*/*MS* analysis of trypsinized peptides from the gel spot identified that 24 peptide fragments were derived from protein SPICI03_175, the alpha subunit of pyruvate dehydrogenase E1 subunit (Sequest score, 583; sequence coverage, 67 %), and that 16 peptide fragments were derived from protein SPICI01B_002, the beta chain of DNA polymerase III (Sequest score, 99; sequence coverage, 46 %). In *M. pneumoniae*, pyruvate dehydrogenase E1 beta component can be surface translocated and binds host fibronectin [44]. Thus in this mycoplasma, pyruvate dehydrogenase E1 component functions in adherence in addition to its biosynthetic activity. Other cases of multifunctional proteins, such as the ABC transporter subunit OppA in *M. hominis* [38] or the phosphoglycerate kinase in *S. citri* [45, 46], involved in both cell metabolism and adhesion have been described in mollicutes. In *S. citri* GII-3 wt, a role of dehydrogenase E1 subunit in adhesion of spiroplasma to insect cells in addition to its role in pyruvate metabolism cannot be ruled out. In this case however, the presence of this subunit would not be essential for efficient transmission of *S. citri* by its vector.

## Conclusions

Host specialization by bacterial pathogens requires a repertoire of virulence factors and dynamic regulation of gene expression. The present study provided us with a snapshot of the spiroplasma’s response to its hosts and offered the opportunity to identify protein candidates required for maintenance and/or virulence in the different hosts for further functional studies. Consistently with the idea that pathogenic bacteria adapt to various host environments by varying synthesis of surface components, several *S. citri* lipoprotein genes were shown to be regulated in *C. haematoceps* and in periwinkles. Spiroplasma ability to regulate gene expression in both hosts is probably at least partially responsible for its capacity to multiply in plant as well as in its leafhopper vector and for its remarkable abilities to survive in a wide range of leafhoppers and plants [1]. In addition, most lipoprotein genes tested were up-regulated in insects compared to plants. It seems that insects are more favorable than plants for the lipoprotein gene expression of *S. citri*. This implies that these lipoprotein genes could be involved in adhesion and/or in invasion of insect cells during the transmission process. In addition to lipoprotein genes, gene encoding the E1 component of pyruvate dehydrogenase represents a good candidate for being involved in spiroplasma adaptation to its vector.

Finally, despite the small size of the *S. citri* chromosome, the regulation at transcription gene level in *S. citri* likely plays a significant role in its adaptive capacities to its hosts and could constitute an efficient mean for shaping the spiroplasma surface in response to the environmental conditions. Transcriptional regulation upon interaction with the host environment seems to be more developed in *S. citri* than in *M. pneumoniae* [39], *M. gallisepticum* [42] or in *M. hyopneumoniae* [43]. It has been suggested that the close adaptation to specific mucosal environments, such as the human lung epithelium for *M. pneumoniae*, was associated to restricted regulating capacities at the gene level [47]. Following this assumption, the important transcriptional regulating capacities in *S. citri* compared to these three mycoplasmas may be associated to the versatile environment (two distinct hosts) encountered by the spiroplasma.

## Availability of data and materials

The data sets supporting the results of this article are included within the article and its additional file. GenBank accession numbers for *S. citri* chromosomal contigs are AM285301-AM285339. Genbank accession numbers for *S. citri* plasmids pSci1 to pSci6 sequences are AJ969069-AJ969074.

## Additional file

Additional file 1: Table S1. Relative lipoprotein genes expression levels (-ΔΔC_T_) in graft-inoculated plants (GIP), in leafhopper-infected plants (LIP) and in insects. (XLSX 10 kb)

## Abbreviations

CTAB: Cetyl trimethyl ammonium bromide
GIP: Graft-inoculated periwinkles
LC-MS/MS: Liquid chromatography-tandem mass spectrometry
LIP: Leafhopper-infected periwinkles
PCR: Polymerase chain reaction
PTS: Phosphoenolpyruvate transferase system
RT-PCR: Reverse transcription-polymerase chain reaction
ScARP: *S. citri* adhesion-related protein

## References

[1] Calavan EC, Bové JM, Whitcomb RF, Tully JG (1989). Ecology of *Spiroplasma citri*. The mycoplasmas.

[2] Fletcher J, Schultz GA, Davis RE, Eastman CE, Goodman RM (1981). Brittle root disease of horseradish: evidence for an etiological role of *Spiroplasma citri*. Phytopathology.

[3] Fletcher J, Wayadande A, Melcher U, Ye FC (1998). The phytopathogenic mollicute-insect vector interface: a closer look. Phytopathology.

[4] Kwon MO, Wayadande AC, Fletcher J (1999). *Spiroplasma citri* movement into the intestines and salivary glands of its leafhopper vector, *Circulifer tenellus*. Phytopathology.

[5] Liu HY, Gumpf DJ, Oldfield GN, Calavan EC (1983). Transmission of *Spiroplasma citri* by *Circulifer tenellus*. Phytopathology.

[6] Gaurivaud P, Danet JL, Laigret F, Garnier M, Bové JM (2000). Fructose utilization and phytopathogenicity of *Spiroplasma citri*. Mol Plant Microbe In.

[7] Liang FT, Nelson FK, Fikrig E (2002). Molecular adaptation of *Borrelia burgdorferi* in the murine host. J Exp Med.

[8] Kovacs-Simon A, Titball RW, Michell SL (2011). Lipoproteins of bacterial pathogens. Infect Immun.

[9] Carle P, Saillard C, Carrere N, Carrere S, Duret S, Eveillard S, Gaurivaud P, Gourgues G, Gouzy J, Salar P (2010). Partial chromosome sequence of *Spiroplasma citri* reveals extensive viral invasion and important gene decay. Appl Environ Microbiol.

[10] Sankaran K, Wu HC (1995). Bacterial prolipoprotein signal peptidase. Methods Enzymol.

[11] Krogh A, Larsson B, von Heijne G, Sonnhammer EL (2001). Predicting transmembrane protein topology with a hidden Markov model: application to complete genomes. J Mol Biol.

[12] Wroblewski H, Johansson KE, Hjérten S (1977). Purification and characterization of spiralin, the main protein of the *Spiroplasma citri* membrane. Biochim Biophys Acta.

[13] Duret S, Berho N, Danet JL, Garnier M, Renaudin J (2003). Spiralin is not essential for helicity, motility, or pathogenicity but is required for efficient transmission of *Spiroplasma citri* by its leafhopper vector *Circulifer haematoceps*. Appl Environ Microbiol.

[14] Killiny N, Castroviejo M, Saillard C (2005). *Spiroplasma citri* spiralin acts in vitro as a lectin binding to glycoproteins from its insect vector *Circulifer haematoceps*. Phytopathology.

[15] Duret S, Batailler B, Dubrana MP, Saillard C, Renaudin J, Béven L, Arricau-Bouvery N (2014). Invasion of insect cells by *Spiroplasma citri* involves spiralin relocalization and lectin/glycoconjugate-type interactions. Cell Microbiol.

[16] Boutareaud A, Danet JL, Garnier M, Saillard C (2004). Disruption of a gene predicted to encode a solute binding protein of an ABC transporter reduces transmission of *Spiroplasma citri* by the leafhopper *Circulifer haematoceps*. Appl Environ Microbiol.

[17] Saillard C, Carle P, Duret-Nurbel S, Henri R, Killiny N, Carrère S, Gouzy J, Bové JM, Renaudin J, Foissac X (2008). The abundant extrachromosomal content of *Spiroplasma citri* strain GII3-3X. BMC Genomics.

[18] Breton M, Duret S, Danet JL, Dubrana MP, Renaudin J (2010). Sequences essential for transmission of *Spiroplasma citri* by its leafhopper vector, *Circulifer haematoceps*, revealed by plasmid curing and replacement based on incompatibility. Appl Environ Microbiol.

[19] Berho N, Duret S, Renaudin J (2006). Absence of plasmids encoding adhesion-related proteins in non insect-transmissible strains of *Spiroplasma citri*. Microbiology.

[20] Duret S, Batailler B, Danet J-L, Béven L, Renaudin J, Arricau-Bouvery N (2010). Infection of the *Circulifer haematoceps* cell line Ciha-1 by *Spiroplasma citri*: the non insect-transmissible strain 44 is impaired in invasion. Microbiology.

[21] Béven L, Duret S, Batailler B, Dubrana MP, Saillard C, Renaudin J, Arricau-Bouvery N (2012). The repetitive domain of ScARP3d triggers entry of *Spiroplasma citri* into cultured cells of the vector *Circulifer haematoceps*. PLoS One.

[22] André A, Maccheroni W, Doignon F, Garnier M, Renaudin J (2003). Glucose and trehalose PTS permeases of *Spiroplasma citri* probably share a single IIA domain, enabling the spiroplasma to adapt quickly to carbohydrate changes in its environment. Microbiology.

[23] Vignault JC, Bové JM, Saillard C, Vogel R, Farro A, Venegas L, Stemmer W, Aoki S, McCoy RE, Al-Beldawi AS (1980). Mise en culture de spiroplasmes à partir de matériel végétal et d’insectes provenant de pays circum méditerranéens et du Proche Orient. C R Acad Sci Ser III.

[24] Tully JG, Whitcomb RF, Clark HF, Williamson DL (1977). Pathogenic mycoplasma: cultivation and vertebrate pathogenicity of a new spiroplasma. Science.

[25] Foissac X, Danet JL, Saillard C, Gaurivaud P, Laigret F, Pare C, Bové JM (1997). Mutagenesis by insertion of Tn*4001* into the genome of *Spiroplasma citri*: characterization of mutants affected in plant pathogenicity and transmission to the plant by the leafhopper vector *Circulifer haematoceps*. Mol Plant Microbe In.

[26] Murray MG, Thompson WF (1980). Rapid isolation of high molecular weight plant DNA. Nucleic Acids Res.

[27] Chevalier C, Saillard C, Bové JM (1990). Organization and nucleotide sequences of the *Spiroplasma citri* genes for ribosomal protein S2, elongation factor Ts, spiralin, phosphofructokinase, pyruvate kinase, and an unidentified protein. J Bacteriol.

[28] Livak KJ, Schmittgen TD (2001). Analysis of relative gene expression data using real-time quantitative PCR and the 2(-Delta Delta C(T)) Method. Methods.

[29] Killiny N, Batailler B, Foissac X, Saillard C (2006). Identification of a *Spiroplasma citri* hydrophilic protein associated with insect transmissibility. Microbiology.

[30] Fairbanks G, Steck TL, Wallach DFH (1971). Electrophoretic analysis of the major polypeptides of human erythrocyte membrane. Biochemistry.

[31] Paredes JC, Herren JK, Schupfer F, Marin R, Claverol S, Kuo CH, Lemaitre B, Beven L (2015). Genome sequence of the Drosophila melanogaster male-killing Spiroplasma strain MSRO endosymbiont. MBio.

[32] Williamson DL, Renaudin J, Bové JM (1991). Nucleotide sequence of the *Spiroplasma citri* fibril protein gene. J Bacteriol.

[33] Davidson AL, Dassa E, Orelle C, Chen J (2008). Structure, fonction and evolution of bacterial ATP-binding cassette systems. Microbiol Mol Biol Rev.

[34] André A, Maucourt M, Moing A, Rolin D, Renaudin J (2005). Sugar import and phytopathogenicity of *Spiroplasma citri*: glucose and fructose play distinct roles. Mol Plant Microbe In.

[35] Becker A, Schlöder P, Steele JE, Wegener G (1996). The regulation of trehalose metabolism in insects. Experimentia.

[36] Thompson SN (2003). Trehalose, the insect “blood” sugar. Adv Insect Physiol.

[37] Gaurivaud P, Laigret F, Garnier M, Bové JM (2001). Characterization of FruR as a putative activator of the fructose operon of *Spiroplasma citri*. FEMS Microbiol Lett.

[38] Hopfe M, Dahlmanns T, Henrich B (2011). In *Mycoplasma hominis* the OppA-mediated cytoadhesion depends on its ATPase activity. BMC Microbiol.

[39] Hallamaa KM, Tang SL, Ficorilli N, Browning GF (2008). Differential expression of lipoprotein genes in *Mycoplasma pneumoniae* after contact with human lung epithelial cells, and under oxidative and acidic stress. BMC Microbiol.

[40] Castano S, Blaudez D, Desbat B, Dufourcq J, Wroblewski H (2002). Secondary structure of spiralin in solution, at the air/water interface, and its interaction with lipid monolayers. Biochim Biophys Acta.

[41] Güell M, van Noort V, Yus E, Chen WH, Leigh-Bell J, Michalodimitrakis K, Yamada T, Arumugam M, Doerks T, Kuhner S (2009). Transcriptome complexity in a genome-reduced bacterium. Science.

[42] Cecchini KR, Gorton TS, Geary SJ (2007). Transcriptional responses of *Mycoplasma gallisepticum* strain R in association with eukaryotic cells. J Bacteriol.

[43] Madsen ML, Puttamreddy S, Thacker EL, Carruthers MD, Minion FC (2008). Transcriptome changes in *Mycoplasma hyopneumoniae* during infection. Infect Immun.

[44] Dallo SF, Kannan TR, Blaylock MW, Baseman JB (2002). Elongation factor Tu and E1 beta subunit of pyruvate dehydrogenase complex act as fibronectin binding proteins in *Mycoplasma pneumoniae*. Mol Microbiol.

[45] Labroussaa F, Arricau-Bouvery N, Dubrana M-P, Saillard C (2010). Entry of *Spiroplasma citri* into *Circulifer haematoceps* cells involves interaction between spiroplasma phosphoglycerate kinase and leafhopper actin. Appl Environ Microbiol.

[46] Labroussaa F, Dubrana MP, Arricau-Bouvery N, Beven L, Saillard C (2011). Involvement of a minimal actin-binding region of *Spiroplasma citri* phosphoglycerate kinase in spiroplasma transmission by its leafhopper vector. PLoS One.

[47] Stülke J, Eilers H, Schmidl SR, Schaechter M (2009). Mycoplasma and spiroplasma. Encyclopedia of Microbiology.

